# Solid-Phase Extraction Based on Captiva EMR-Lipid for Determination of 19 Aromatic Amine Antioxidants and Two *p*-Phenylenediamine Quinones in Human Plasma

**DOI:** 10.3390/toxics14030187

**Published:** 2026-02-24

**Authors:** Bowen Liang, Qing Deng, Zibin Pan, Bibai Du, Lixi Zeng

**Affiliations:** 1 Guangdong Key Laboratory of Environmental Pollution and Health, College of Environment and Climate, Jinan University, Guangzhou 511443, China; liangbowen8867@163.com (B.L.); 17340512415@163.com (Q.D.); johnperry_pan@163.com (Z.P.); lxzeng@jnu.edu.cn (L.Z.); 2 School of Information Engineering, Guangdong Meizhou Vocational and Technological College, Meizhou 514011, China; 3 College of Resources and Environmental Engineering, Guizhou University, Guiyang 550025, China

**Keywords:** antioxidant, new pollutant, lipid removal, solid-phase extraction (SPE), human blood

## Abstract

A robust analytical method based on Captiva EMR-Lipid solid-phase extraction and HPLC-MS/MS was developed and validated for the simultaneous determination of 19 aromatic amine antioxidants (AAs) and two *p*-phenylenediamine-derived quinones (PPD-Qs) in human plasma. The optimized protocol effectively removed phospholipid interferences from complex blood matrix, significantly mitigating ion suppression and improving the recovery of hydrophobic AAs compared to conventional liquid–liquid extraction. Method validation demonstrated good accuracy (spike recoveries: 73.0–96.8%), precision (RSD < 11%), and sensitivity with method detection limits ranging from 0.81 to 21 pg/mL. The method was successfully applied to plasma samples from 20 adults, in which 11 AAs were detected at total concentrations of 240–710 pg/mL. Diphenylamine derivatives, particularly bis(4-tert-butylphenyl)amine (DBDPA) and diphenylamine (DPA), were identified as the predominant compounds, contributing over 69% of the total AA burden. No PPDs or PPD-Qs were detected, which may be attributed to their biotransformation and urinary excretion, as well as the limited sample size. This study provides a comprehensive biomonitoring tool for assessing combined human exposure to multiple AAs and establishes a foundation for further investigation into their health implications.

## 1. Introduction

Aromatic amine antioxidants (AAs) are a class of chemical additives widely used in industrial and consumer goods sectors, primarily to prevent oxidative degradation of polymer materials (e.g., rubber, plastics) and lubricants caused by heat, oxygen, and light, thereby extending product life and maintaining performance stability [1]. Based on their specific chemical structures, AAs are classified into diphenylamines (DPAs), *p*-phenylenediamines (PPDs), and naphthylamines (NPAs). Currently, several AAs such as 4,4′-bis(phenylisopropyl)diphenylamine (diAMS), N-phenyl-1-naphthylamine (PANA), and N-(1,3-dimethylbutyl)-N′-phenyl-*p*-phenylenediamine (6PPD) are listed as High-Production-Volume chemicals by the U.S. Environmental Protection Agency [2]. With increasing market demand in rubber, plastics, and related fields, the annual production and consumption of AAs continue to grow [3]. However, substantial evidence indicates that these chemicals pose non-negligible potential health risks. Some AAs and their transformation products have been confirmed to exhibit aquatic lethal toxicity, developmental toxicity, mammalian organ toxicity, and neurotoxicity, among others [4,5,6,7,8]. A prominent example is the oxidation product of 6PPD, 6PPD-quinone (6PPD-Q), identified as a key toxicant causing acute mortality in coho salmon [9]. In the ECHA industry classifications, it is labeled as H400, indicating that it is very toxic to aquatic life [10]. In early-life stage tests with fathead minnows, both DPA and PANA caused teratogenic and lethal effects, with lethality being the more sensitive endpoint [11]. In a study on wild mammals, the toxicity of DPA was primarily targeted to the kidneys, leading to reduced urinary concentrating capacity and potentially resulting in dehydration and anemia. Additionally, reproductive and developmental impairments were observed across multiple species [7]. Consequently, with rising production and use, the potential threats of AAs to ecosystems and human health are attracting increasing attention in environmental science and public health research.

Through industrial production and daily consumption activities, AAs can enter the environment via multiple pathways, including product wear, waste disposal, and others [12,13]. Recent environmental monitoring studies have detected AAs in various environmental media, such as atmospheric particulate matter, surface water, sediments, soil, and indoor/outdoor dust [14,15,16,17]. More notably, these compounds are not only present in the external environment but have also been shown to enter the human body through multiple routes—inhalation, diet, dermal contact—resulting in internal exposure. Numerous biomonitoring studies have detected various AAs and their transformation products in human samples, including urine, blood, breast milk, and even cerebrospinal fluid [18,19,20,21,22], providing direct evidence of human body burden and internal translocation. Human blood, as a key medium for the transport, distribution, and metabolism of chemicals within the body, can more comprehensively reflect recent and long-term exposure levels of individuals, serving as an important biomarker for health risk assessment [23].

Current research on detecting AAs in human blood primarily focuses on a few high-concern PPD compounds, such as 6PPD and its transformation product 6PPD-Q. Analytical methods predominantly employ high-performance liquid chromatography-tandem mass spectrometry (HPLC-MS/MS), which has become a mainstream technique for trace organic pollutant analysis due to its high sensitivity and selectivity [24,25,26]. However, existing studies on the analysis of AAs in blood often employ relatively simplistic sample pretreatment procedures for blood, such as protein precipitation followed by direct injection, or only basic steps like liquid–liquid extraction (LLE) or freeze-lipid removal [19,20,22,27], lacking in-depth purification tailored to the complex blood matrix. The human blood matrix is highly complex, containing substantial endogenous substances such as proteins, phospholipids, cholesterol, and fatty acids. These matrix components, especially phospholipids, can cause significant ion suppression or enhancement effects in electrospray ionization-mass spectrometry analysis, severely interfering with accurate identification and quantification of target compounds, and potentially leading to instrument contamination and reduced sensitivity [28].

Therefore, developing a sample pretreatment method for blood that efficiently removes interfering substances such as phospholipids while enabling simultaneous accurate determination of multiple AAs and their important transformation products is crucial for comprehensively assessing human exposure levels and related health risks. Solid-phase extraction (SPE), offering advantages such as low solvent consumption, high clean-up efficiency, and simple operation, has become a mainstream pretreatment technique for the analysis of trace organic compounds in complex biological matrices. The novel enhanced matrix removal–lipid SPE sorbent (Captiva EMR-Lipid) selectively adsorbs and efficiently removes lipids from samples through a mechanism combining size exclusion and hydrophobic interactions with the long aliphatic chains of lipids, while maximizing the recovery of target analytes. Building on this, we developed and optimized a method integrating Captiva EMR-Lipid SPE with HPLC-MS/MS, achieving for the first time simultaneous extraction, purification, and determination of 19 AAs (covering DPAs, PPDs, and NPAs) and 2 key PPD-derived quinones (PPD-Qs). The established method addresses gaps in existing approaches, significantly improving analytical accuracy and precision. It provides a robust analytical tool for the systematic and comprehensive assessment of combined human exposure to multiple AAs, laying a solid methodological foundation for future large-scale population biomonitoring and in-depth toxicological risk research.

## 2. Materials and Methods

### 2.1. Chemicals and Reagents

The target analytes in this study encompass 19 commercially prevalent AAs, including DPAs, PPDs, and NPAs, as well as 2 PPD-Qs. A complete list of the target compounds, including their abbreviations, CAS numbers, chemical structures, and basic physicochemical properties, is provided in Table A1. Authentic standards were purchased from Tokyo Chemical Industry (Tokyo, Japan), J&K Scientific (Beijing, China), AccuStandard (New Haven, CT, USA), and Cambridge Isotope Laboratories (Andover, MA, USA), with purities > 98%. Methanol and acetonitrile (HPLC grade) used in the experiments were obtained from Fisher Scientific (Fair Lawn, NJ, USA). Ultrapure water was prepared using a Milli-Q Integral system. Captiva EMR-Lipid (3 mL, 300 mg) for SPE was purchased from Agilent Technologies (Santa Clara, CA, USA).

### 2.2. Sample Collection

Blood samples were collected from 20 randomly recruited volunteers at Zhujiang Hospital of Southern Medical University in 2020. Approximately 10 mL of blood was drawn from each volunteer using vacuum blood collection tubes. Each blood sample was immediately centrifuged thoroughly at 4 °C to separate plasma from red blood cells. The resulting plasma samples were stored at −80 °C until chemical analysis. The study protocol was approved by the ethics committee of Jinan University, and written informed consent was obtained from all participants.

### 2.3. Standard Solutions

Twenty-one standard compounds were accurately weighed and dissolved in methanol, acetonitrile, or other appropriate solvents to prepare individual primary stock solutions at a concentration of 10 mg/mL. These primary stock solutions were then diluted with methanol to obtain secondary stock solutions at 100 μg/mL. Aliquots from each secondary stock solution were mixed and further diluted with acetonitrile to prepare a mixed standard solution at 1 μg/mL. The mixed standard solution was serially diluted to prepare a calibration curve at concentrations of 100, 50, 10, 5, 1, 0.5, 0.1, 0.05, and 0.01 ng/mL.

### 2.4. Sample Pretreatment

LLE protocol: 5 ng of each standard was spiked into 0.5 mL of plasma and allowed to equilibrate. Subsequently, 2 mL of acetonitrile and 0.2 g of NaCl were added to induce protein precipitation and phase separation. The mixture was vortexed, sonicated for 20 min, and centrifuged. The supernatant was collected, and the extraction was repeated twice. The combined supernatants were then treated with 20 μL of 1 mmol/L glutathione to prevent oxidation of PPDs, concentrated to 0.5 mL under a gentle stream of nitrogen, and analyzed.

Captiva EMR-Lipid clean-up: To each 0.5 mL plasma sample, 2 mL of acetonitrile was added for ultrasound-assisted extraction. After centrifugation, the supernatant was loaded onto a Captiva EMR-Lipid SPE cartridge. The cartridge was then eluted with 2.5 mL of acetonitrile-water mixture (4:1, *v*/*v*). The combined loading solution and eluate were collected, followed by the addition of 0.4 g NaCl. After vortex mixing and centrifugation, the upper acetonitrile layer was transferred. Glutathione (20 μL, 1 mmol/L) was added, and the solution was concentrated under a gentle stream of nitrogen to 0.5 mL. Finally, the extract was filtered through a 0.22 μm PTFE filter prior to analysis.

### 2.5. Instrument Analysis

**Figure 1:**
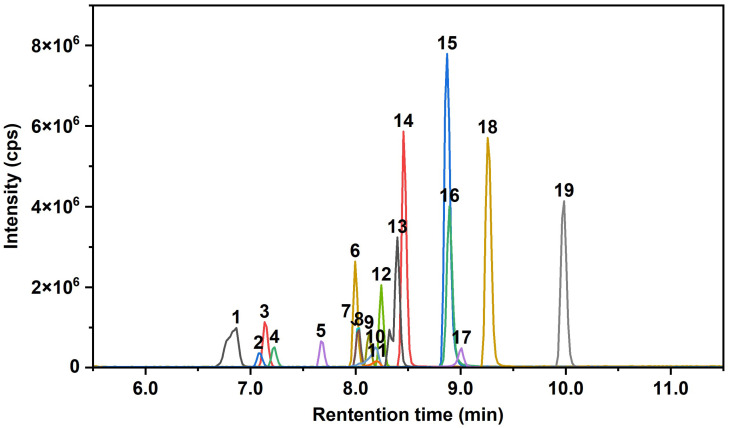
HPLC-ESI (+)-MS/MS multiple reaction monitoring (MRM) chromatograms of a standard mixture containing the 19 AAs and 2 PPD-Qs (10 ng/mL for each). 1. IPPD, 2. CPPD, 3. 6PPD, 4. AW, 5. IPPD-Q, 6. DPA, 7. 4NO_2_-DPA, 8. 6PPD-Q, 9. 2NO_2_-DPA, 10. 24NO_2_-DPA, 11. DPPD, 12. DM-AD, 13. PANA/PBNA, 14. BDPA, 15. DBDPA, 16. DPB, 17. DNPD, 18. diAMS, 19, DNODPA/DTODPA.

Nineteen AAs and two PPD-Qs were quantified using an ExionLC AC high-performance liquid chromatography system coupled with a Triple Quad 5500 mass spectrometer (AB SCIEX, Framingham, MA, USA). Chromatographic separation was performed on an XBridge BEH C8 column (2.5 μm, 2.1 mm × 100 mm, Waters, Milford, MA, USA) with an injection volume of 5 μL. The column temperature was maintained at 40 °C. The mobile phase consisted of 0.1% formic acid in water (A) and methanol (B). The elution gradient was programmed as follows, 0–1.5 min, 10% B; 4–15 min, 100% B; and 15.1–20 min, 10% B, at a flow rate of 0.3 mL/min. To minimize contamination of the mass spectrometer, a six-port valve was used to divert the effluent to waste during periods when no target analytes were eluting. The mass spectrometer was operated in positive electrospray ionization (ESI) mode with multiple reaction monitoring (MRM). The ion spray voltage was set to 5500 V, the ion source temperature to 550 °C, the curtain gas pressure to 40 psi, and both the nebulizer gas and auxiliary heater gas pressures to 40 psi. Optimized MS/MS parameters for all target analytes are summarized in Table 1, and their representative chromatograms are presented in Figure 1.

### 2.6. Validation Procedure

The proposed method was validated in terms of accuracy, precision, and matrix effects, while its linearity, limit of detection (LOD), and limit of quantification (LOQ) were also evaluated. Method accuracy was assessed via procedural blanks and spike recovery experiments. Procedural blanks were prepared by replacing plasma samples with ultrapure water and subjecting them to the same pretreatment process. Spike recovery was calculated as follows: Recovery (%) = [(measured concentration in spiked sample − measured concentration in unspiked sample)/spiked concentration] × 100%. Precision was evaluated as the relative standard deviation (RSD) of the spike recovery results. Matrix effect (ME) was calculated as: ME (%) = [(measured concentration in post-extraction spiked sample − measured concentration in unspiked sample)/spiked concentration] × 100%. The spiking concentration was 5000 pg/mL. Method linearity was evaluated based on the coefficient of determination (*R*^2^) obtained from linear regression of calibration curves within the concentration range of 0.01–100 ng/mL. The LOD and LOQ were defined as the concentrations corresponding to signal-to-noise ratios of 3 and 10, respectively. If a target analyte was detected in procedural blanks, the LOD and LOQ were alternatively set to 3 times and 10 times the standard deviation of the blank measurements, respectively.

### 2.7. Data Analysis

Chromatographic peaks were integrated using Analyst 1.6.3 (AB SCIEX), and the corresponding peak areas were applied to the calibration curves for quantifying target analytes. DNODPA and DTODPA, as well as PANA and PBNA, are isomeric compounds that share identical precursor and product ions in mass spectrometry and could not be chromatographically separated. Consequently, they were quantified collectively. Statistical analysis was conducted using SPSS 27 (IBM Corporation, Armonk, NY, USA). For analyte concentrations measured below the LOD and LOQ, the values were substituted with LOD (or LOQ) multiplied by the detection frequency (DF).

## 3. Results and Discussion

### 3.1. Optimization of Instrument Conditions

In our previous study, the chromatographic and mass spectrometric conditions for the 21 target AAs and PPD-Qs were systematically optimized [13]. The chromatographic optimization included the selection of mobile phase composition, type and concentration of mobile phase additives, column type, and gradient elution profile. Mass spectrometric optimization encompassed ion source parameters (such as spray voltage, ion source temperature, nebulizer gas, heater gas, and curtain gas) and compound-dependent parameters (including collision energy and declustering potential). Based on the established method, the present study further introduced the use of a six-port switching valve to control the chromatographic effluent, allowing only the eluent within the retention time window of the target analytes to enter the mass spectrometer. This configuration effectively prevents highly hydrophilic substances, such as salts, from entering and contaminating the mass spectrometer.

### 3.2. Optimization of Pre-Treatment Method

**Figure 2:**
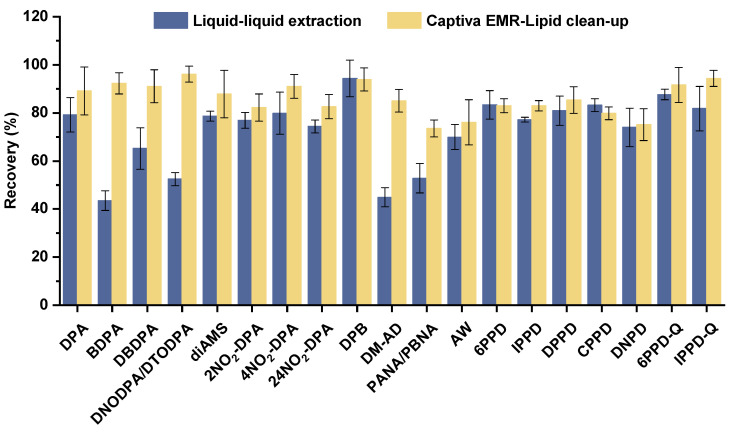
Plasma spiked recovery rates (mean ± SD, *n* = 3) of 19 AAs and 2 PPD-Qs under liquid–liquid extraction and Captiva EMR-Lipid clean-up.

The spike recoveries of 19 AAs and 2 PPD-Qs were evaluated using an LLE protocol. As shown in Figure 2, under the LLE-only procedure, the recoveries of all target analytes ranged from 43.5% to 94.4%. Among them, BDPA, DNODPA/DTODPA, DM-AD, and PANA/PBNA exhibited recoveries below 60%. After excluding insufficient extraction efficiency or significant analyte loss during the process, the low recoveries were likely attributable to ion suppression caused by lipids in the blood matrix during mass spectrometric analysis. This hypothesis was further supported by the relatively higher recoveries (ranging from 74.0% to 87.7%) observed for the more hydrophilic PPDs and PPD-Qs, which tend not to co-elute with lipids in the chromatographic separation, and are therefore generally less susceptible to lipid interference [29,30].

To achieve satisfactory recoveries for all target analytes in practical analyses, it was necessary to introduce a cleanup step prior to instrumental analysis. The extraction protocol was therefore modified as follows: after adding acetonitrile to the plasma sample to induce protein precipitation, the mixture was loaded onto a Captiva EMR-Lipid SPE cartridge for targeted lipid removal. Subsequent phase separation and concentration were performed using the same procedure described above. With this improvement, the recoveries of the previously low-recovery analytes increased significantly, with mean values all exceeding 70%. The overall recovery range for all analytes improved to 73.5–96.1%. These results demonstrate that the removal of lipid during the Captiva EMR-Lipid SPE process effectively mitigated ion suppression caused by blood matrix without introducing substantial analyte loss. Consequently, the inclusion of Captiva EMR-Lipid SPE as a clean-up step in plasma sample pretreatment was adopted, and further validation of this optimized method was carried out.

### 3.3. Method Validation

**Figure A1:**
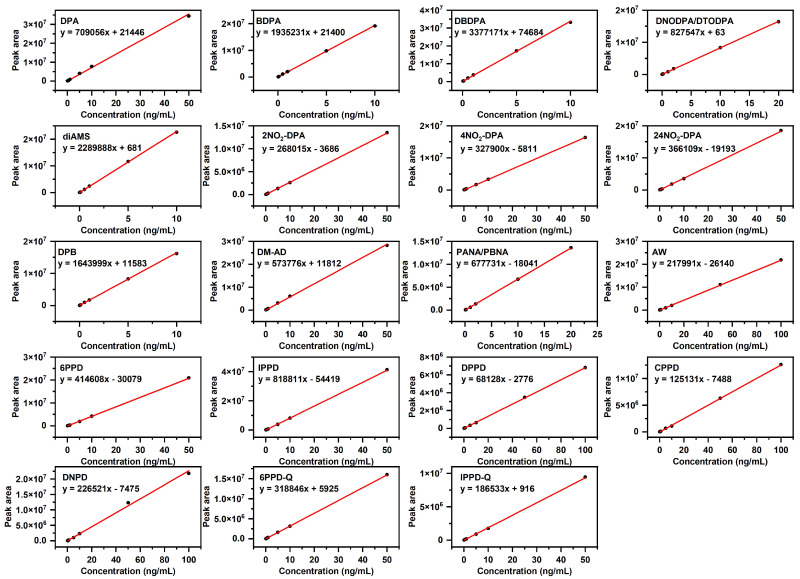
Calibration curves for the 19 AAs and 2 PPD-Qs.

A comprehensive methodological validation was performed for the established analytical method, which combines Captiva EMR-Lipid cleanup with HPLC-MS/MS for the determination of 19 AAs and 2 PPD-Qs in human plasma. Table 2 summarizes the procedural blanks introduced during sample preparation, the recoveries at spiked levels of 5000 pg/mL and 2500 pg/mL, and the matrix effects for all target analytes (*n* = 3). No target analytes were detected in the procedural blanks. At both spiking levels, the recoveries of AAs and PPDs ranged from 73.0% to 96.8%, with relative standard deviations (RSDs) of recovery between 2.6% and 11%, demonstrating acceptable accuracy and precision of the method. The matrix effects for the target analytes fell within 74.4% to 93.7%, further indicating that matrix interference was effectively mitigated. The linearity parameters of the calibration curves for the 19 AAs and 2 PPD-Qs, as well as the LODs and LOQs, are presented in Table 3. All calibration curves exhibited *R*^2^ ≥ 0.996 (Figure A1), indicating good linearity across their linear ranges. The LODs and LOQs ranged from 0.813 to 20.9 pg/mL and from 2.71 to 69.7 pg/mL, respectively. Compared with previously reported methods, the developed method successfully incorporated DPAs and NPAs in addition to PPDs and PPD-Qs. These compounds have been overlooked in human exposure studies, and the method achieved satisfactory recoveries and comparable LOQs (Table 4). These findings indicate the potential of the established method for analyzing trace levels of AAs and PPDs in plasma.

### 3.4. Actual Plasma Sample Detection

**Figure 3:**
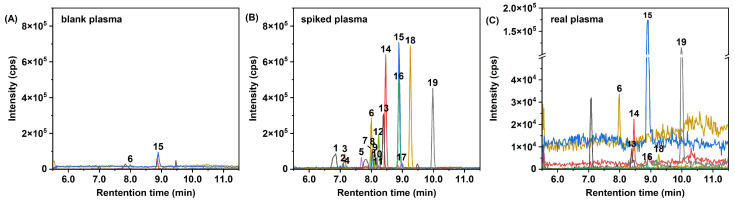
HPLC-ESI(+)-MS/MS multiple reaction monitoring (MRM) chromatograms of (**A**) blank plasma, (**B**) spiked plasma (spiking level: 1 ng/mL) and (**C**) real plasma. 1. IPPD, 2. CPPD, 3. 6PPD, 4. AW, 5. IPPD-Q, 6. DPA, 7. 4NO_2_-DPA, 8. 6PPD-Q, 9. 2NO_2_-DPA, 10. 24NO_2_-DPA, 11. DPPD, 12. DM-AD, 13. PANA/PBNA, 14. BDPA, 15. DBDPA, 16. DPB, 17. DNPD, 18. diAMS, 19, DNODPA/DTODPA.

**Figure A2:**
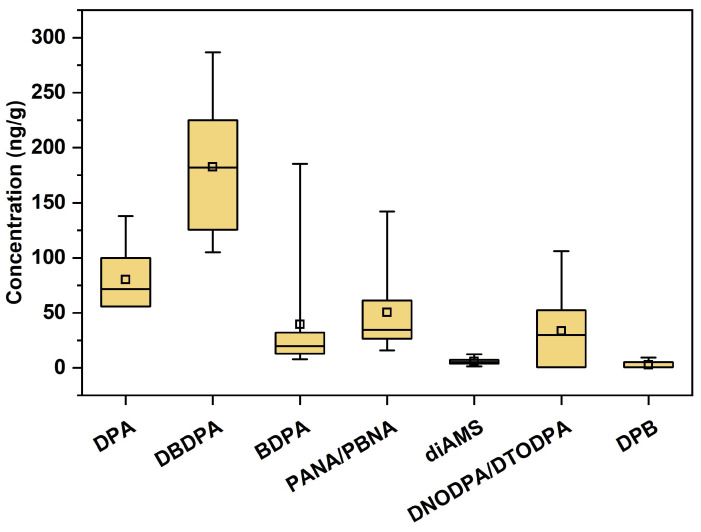
Boxplot of the concentration distributions of the nine quantified AAs in plasma. The boxes represent the 25th, 50th, and 75th percentiles, respectively. Squares indicate the mean values. The whiskers denote the 5th and 95th percentiles, respectively.

The established method was applied to the analysis of plasma samples collected from 20 general adults. Figure 3 presents the MRM chromatograms of blank plasma, spiked plasma, and a representative real plasma sample. Eleven AAs were detected, and the statistical results and distributions of their concentrations are presented in Table 5 and Figure A2. The total concentration of the 11 detected AAs in plasma ranged from 240 to 710 pg/mL, with a median concentration of 400 pg/mL. DPA, DBDPA, BDPA, PANA/PBNA, diAMS, and DNODPA/DTODPA were frequently detected in the samples, with detection frequencies (DF) > 60%; among these, DPA and DBDPA were detected in all samples. DBDPA was the predominant AA in plasma, with a median concentration of 180 pg/mL and an average contribution of 47.2%, followed by DPA (21.9%), PANA/PBNA (11.8%), and DNODPA/DTODPA (8.6%). No PPDs or PPD-Qs were detected, which is consistent with previously reported low detection rates of PPDs and PPD-Qs in blood [19]. This observation can be attributed to their biotransformation in vivo and excretion via urine [18,22]. While this study is limited by its small sample size, these results demonstrate that the general adult population is likely widely exposed to multiple AAs, with DPAs which have received relatively little attention, contributing to a non-negligible internal burden. The capability of the present method to analyze a broad spectrum of AAs can provide a more comprehensive assessment of combined human exposure than previous methods that primarily targeted PPD-related compounds.

## 4. Conclusions

In this study, we observed that when the LLE method commonly used for analyzing PPDs and PPD-Qs in blood was applied to AAs including DPAs, NPAs, and PPDs, the lack of a cleanup step resulted in significant ion suppression during mass spectrometric analysis due to matrix interference, particularly for several hydrophobic AAs. To address this, a robust and sensitive analytical method based on Captiva EMR-Lipid SPE cleanup combined with HPLC-MS/MS was developed and validated for the simultaneous determination of 19 AAs and 2 PPD-Qs in human plasma. The method was successfully applied to plasma samples from a general adult population, leading to the detection of 11 AAs. This work provides a validated tool for biomonitoring multiple AAs and PPD-Qs in human blood, offers preliminary insights into the internal exposure profile of AAs in the general population, and establishes a methodological foundation for future large-scale studies aimed at assessing associated health risks and elucidating human exposure pathways.

## Figures and Tables

**Table 1:** Optimized ESI (+)-MS/MS parameters for 19 AAs and 2 PPD-Qs.

Analyte	Quantitative Transition (CE ^1^, eV)	Confirmation Transition (CE, eV)	DP ^2^ (eV)	RT ^3^ (min)
DPA	170.1 > 93.1 (35)	170.1 > 66.1 (56)	80	8.00
BDPA	226.2 > 134.1 (37)	226.2 > 93.0 (56)	80	8.46
DBDPA	282.2 > 134.2 (46)	282.2 > 106.0 (69)	120	8.89
DNODPA	394.3 > 134.3 (53)	394.3 > 322.3 (55)	110	9.98
DTODPA	394.3 > 134.3 (53)	394.3 > 322.3 (55)	110	9.98
diAMS	406.2 > 196.3 (54)	406.2 > 91.0 (85)	80	9.26
2NO_2_-DPA	215.1 > 180.1 (26)	215.1 > 215.0 (10)	95	8.13
4NO_2_-DPA	215.1 > 198.0 (21)	215.1 > 167.0 (44)	70	8.02
24NO_2_-DPA	260.1 > 167.1 (41)	260.1 > 139.1 (45)	160	8.19
DPB	337.2 > 260.1 (38)	337.2 > 245.1 (34)	95	8.88
DM-AD	210.1 > 194.1 (30)	210.1 > 210.0 (12)	90	8.24
PANA	220.1 > 143.2 (44)	220.1 > 115.0 (65)	120	8.39
PBNA	220.1 > 143.2 (44)	220.1 > 115.0 (65)	120	8.39
AW	218.2 > 160.1 (46)	218.2 > 174.1 (40)	90	7.22
6PPD	269.2 > 184.1 (28)	269.2 > 107.1 (62)	70	7.14
IPPD	227.1 > 184.2 (24)	227.1 > 212.2 (34)	120	6.86
DPPD	261.1 > 168.2 (55)	261.1 > 184.2 (32)	90	8.21
CPPD	267.2 > 185.0 (35)	267.2 > 93.1 (59)	80	7.09
DNPD	361.2 > 218.1 (57)	361.2 > 234.3 (48)	100	9.01
6PPD-Q	299.2 > 241.1 (35)	299.2 > 215.1 (27)	60	8.03
IPPD-Q	257.1 > 215.1 (24)	257.1 > 187.1 (30)	100	7.68

^1^ CE: collision energ. ^2^ DP: declustering potential. ^3^ RT: retention time.

**Table 2:** Procedural blanks, spike recoveries, matrix effects (MEs), and their relative standard deviations (RSDs) for the 19 AAs and 2 PPD-Qs obtained under the extraction procedure incorporating Captiva EMR-Lipid clean-up.

Analyte	Blank (pg/mL)	Recovery (%)	RSD (%)	Recovery (%)	RSD (%)	ME (%)	RSD (%)
		Spike Level (5000 pg/mL)		Spike Level (2500 pg/mL)			
DPA	ND ^1^	89.2	11	88.0	4.9	87.2	8.6
BDPA	ND	92.3	4.8	90.1	4.0	91.3	1.4
DBDPA	ND	91.1	7.5	90.7	11	88.8	6.0
DNODPA/DTODPA	ND	96.1	3.5	96.8	4.7	93.7	6.5
diAMS	ND	87.8	11	88.1	3.7	88.0	3.7
2NO_2_-DPA	ND	82.3	6.9	82.5	8.1	87.6	6.9
4NO_2_-DPA	ND	91.1	5.4	88.8	4.8	86.8	3.7
24NO_2_-DPA	ND	82.6	6.1	79.1	3.5	80.4	6.2
DPB	ND	93.9	5.1	91.2	11	91.0	3.5
DM-AD	ND	85.0	5.5	84.0	3.6	87.9	2.5
PANA/PBNA	ND	73.5	4.8	74.7	5.0	80.1	9.1
AW	ND	76.1	6.2	73.0	8.8	82.8	2.4
6PPD	ND	83.0	3.5	84.2	4.2	89.5	7.2
IPPD	ND	83.0	2.6	85.2	3.1	80.8	5.3
DPPD	ND	85.3	6.5	83.5	5.4	79.7	6.4
CPPD	ND	79.8	3.3	80.7	9.7	82.4	7.6
DNPD	ND	75.1	8.8	79.4	9.2	74.4	6.3
6PPD-Q	ND	91.7	7.9	90.8	5.9	91.1	3.5
IPPD-Q	ND	94.4	3.5	90.5	3.8	90.1	3.0

^1^ ND: not detectable.

**Table 3:** Linear parameters, limits of detection (LODs), and limits of quantification (LOQs) of the established method for the 19 AAs and 2 PPD-Qs.

Analyte	Linear Range (ng/mL)	*R* ^2^	LOD (pg/mL)	LOQ (pg/mL)
DPA	0.1–50	0.997	17	56
BDPA	0.05–10	0.999	3.8	13
DBDPA	0.05–10	0.999	10	34
DNODPA/DTODPA	0.02–20	0.999	0.81	2.7
diAMS	0.01–10	0.999	1.3	4.4
2NO_2_-DPA	0.1–50	0.999	21	70
4NO_2_-DPA	0.1–50	0.999	6.1	20
24NO_2_-DPA	0.1–50	0.999	19	64
DPB	0.01–10	0.999	1.2	4.1
DM-AD	0.1–50	0.999	19	62
PANA/PBNA	0.1–20	0.999	7.8	26
AW	0.1–100	0.998	18	60
6PPD	0.1–50	0.999	7.2	24
IPPD	0.1–50	0.999	18	60
DPPD	0.1–100	0.999	21	68
CPPD	0.1–100	0.999	17	56
DNPD	0.1–100	0.996	14	48
6PPD-Q	0.1–50	0.999	16	53
IPPD-Q	0.1–50	0.999	16	52

**Table 4:** Comparison of the current work with previous methods for analysis of the amine antioxidant-related chemicals.

Matrix	Pretreatment	Instrument	Analyte	Recoveries (%)	LOQs (pg/mL)	Ref.
Whole blood	Freeze-lipid removal	LC-Q-Orbitrap HRMS	Six PPD-Qs	68.8−97.5	19.7−137	[20]
Plasma	LLE	LC-MS/MS	6PPD and 6PPD-Q	63 and 73	40 and 40	[19]
Serum	LLE	LC-MS/MS	Five PPD-Qs	77.8−80.2	2−26	[22]
Serum	LLE	LC-MS/MS	Two PPDs and two PPD-Qs	72−121	1−10	[27]
Plasma	LLE; SPE	LC-MS/MS	19 AAs and two PPD-Qs	73.0−96.8	2.7−70	This study

**Table 5:** Concentrations of AAs and PPD-Qs in 20 plasma samples from general adult (pg/mL).

Analyte	DF ^1^ (%)	GM ^2^	Median	Mean	Range	AP ^3^ (%)
DPA	100	76	72	80	56−140	21.9
DBDPA	100	170	180	180	100−290	47.2
BDPA	95	23	20	40	<MQL − 200	8.3
PANA/PBNA	95	40	35	50	<MQL − 150	11.8
diAMS	90	5.0	5.3	5.9	<MQL − 13	1.5
DNODPA/DTODPA	65	9.5	30	34	<MQL − 130	8.6
DPB	45	<MQL	<MQL	<MQL	<MQL − 9.5	0.8
2NO_2_-DPA	5	<MQL	<MQL	<MQL	<MQL	−
4NO_2_-DPA	5	<MQL	<MQL	<MQL	<MQL	−
∑_11_AAs	−	380	400	380	240−710	−

^1^ DF: detection frequency. ^2^ GM: geometric mean. ^3^ AP: average proportion.

## Data Availability

The original contributions presented in this study are included in the article. Further inquiries can be directed towards the corresponding author.

## Appendix A

**Table A1:** Full names, abbreviations, chemical structures, CAS numbers, molecular weight (MW), and basic physicochemical properties of 19 AAs and 2 PPD-Qs.

Full Name	Abbreviation	Chemical Structure	CAS NO.	MW	LogKow ^1^
Diphenylamine	DPA		122-39-4	169.23	3.50
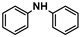
4-tert-Butyldiphenylamine	BDPA		4496-49-5	225.34	5.20
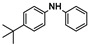
Bis(4-tert-butylphenyl)amine	DBDPA		4627-22-9	281.44	7.11
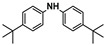
4,4′-Dioctyldiphenylamine	DNODPA		101-67-7	393.66	11.26
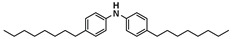
4,4′-Di-tert-octyldiphenylamine	DTODPA		15721-78-5	393.66	10.82
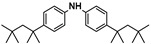
4,4′-Bis(1,1-dimethylbenzyl) diphenylamine	diAMS		10081-67-1	405.59	8.51
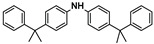
2-Nitrodiphenylamine	2NO_2_-DPA		119-75-5	214.22	3.66
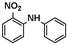
4-Nitrodiphenylamine	4NO_2_-DPA		836-30-6	214.22	3.74
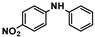
2,4-Dinitrodiphenylamine	24NO_2_-DPA		961-68-2	259.22	3.50
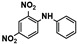
Diphenylbenzidine	DPB		531-91-9	336.44	6.35
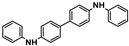
9,9-Dimethylacridan	DM-AD		6267-02-3	209.29	4.14
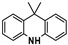
N-Phenyl-1-naphthylamine	PANA		90-30-2	219.29	4.20
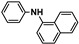
N-Phenyl-2-naphthylamine	PBNA		135-88-6	219.29	4.38
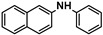
6-Ethoxy-2,2,4-trimethyl-1,2-dihydroquinoline	AW		91-53-2	217.31	3.87
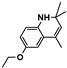
N-(1,3-Dimethylbutyl)-N′-phenyl-p-phenylenediamine	6PPD		793-24-8	268.40	4.68
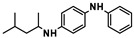
N-Isopropyl-N′-phenyl-p-phenylenediamine	IPPD		101-72-4	226.32	3.28
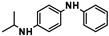
N,N′-Diphenyl-p-phenylenediamine	DPPD		74-31-7	260.34	4.04
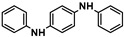
N-Cyclohexyl-N-phenyl-benzene-1,4-diamine	CPPD		101-87-1	266.39	4.64
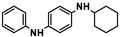
N,N′-Di-2-naphthyl-p-phenylenediamine	DNPD		93-46-9	360.46	6.39
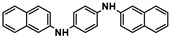
2-[(1,3-Dimethylbutyl)amino]-5-(phenylamino)-2,5-cyclohexadiene-1,4-dione	6PPD-Q		2754428-18-5	298.39	3.98
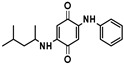
2-[(1-Methylethyl)amino]-5-(phenylamino)-2,5-cyclohexadiene-1,4-dione	IPPD-Q		68054-73-9	256.31	2.58
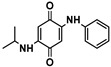

^1^ The logK_ow_ values were predicted by EPI Suite 4.1.
